# How is women’s demand for caesarean section measured? A systematic literature review

**DOI:** 10.1371/journal.pone.0213352

**Published:** 2019-03-06

**Authors:** Clémence Schantz, Myriam de Loenzien, Sophie Goyet, Marion Ravit, Aurélien Dancoisne, Alexandre Dumont

**Affiliations:** 1 CEPED, IRD, Université Paris Descartes, Inserm, équipe SAGESUD, Paris, France; 2 Independent researcher, Annecy Le Vieux, France; 3 French Authority for Health, Saint-Denis La Plaine, France; Univesity of Iowa, UNITED STATES

## Abstract

**Background:**

Caesarean section rates are increasing worldwide, and since the 2000s, several researchers have investigated women’s demand for caesarean sections.

**Question:**

The aim of this article was to review and summarise published studies investigating caesarean section demand and to describe the methodologies, outcomes, country characteristics and country income levels in these studies.

**Methods:**

This is a systematic review of studies published between 2000 and 2017 in French and English that quantitatively measured women’s demand for caesarean sections. We carried out a systematic search using the Medline database in PubMed.

**Findings:**

The search strategy identified 390 studies, 41 of which met the final inclusion criteria, representing a total sample of 3 774 458 women. We identified two different study designs, i.e., cross-sectional studies and prospective cohort studies, that are commonly used to measure social demand for caesarean sections. Two different types of outcomes were reported, i.e., the preferences of pregnant or non-pregnant women regarding the method of childbirth in the future and caesarean delivery following maternal request. No study measured demand for caesarean section during the childbirth process. All included studies were conducted in middle- (n = 24) and high-income countries (n = 17), and no study performed in a low-income country was found.

**Discussion:**

Measuring caesarean section demand is challenging, and the structural violence leading to demand for caesarean section during childbirth while in the labour ward remains invisible. In addition, the caesarean section demand in low-income countries remains unclear due to the lack of studies conducted in these countries.

**Conclusion:**

We recommend conducting prospective cohort studies to describe the social construction of caesarean section demand. We also recommend conducting studies in low-income countries because demand for caesarean sections in these countries is rarely investigated.

## Introduction

Caesarean section (c-section) rates are increasing worldwide. According to two iterative surveys involving 259 facilities and 20 countries, the overall c-section rate increased from 26.4% in 2004–2008 to 31.2% in 2010–11; furthermore, c-section rates have increased in all countries except Japan [1]. The increasing c-section rate is a multifactorial phenomenon. Indeed, technological, professional, legal, ethical, and cultural factors have contributed to the rise in c-sections in the past few decades and have been extensively studied, with a growing consensus that clinical factors alone cannot explain the observed increases [2].

Since the 2000s, several researchers have questioned whether maternal requests for caesarean delivery might explain the increasing c-section rates or whether these rates may be exclusively influenced by midwives and obstetricians [3,4]. Although c-section rates are a reflection of both medical offers and social demand [5], opting for a surgical delivery is not a simple choice [6,7], and the imbalance in power between women and doctors must be considered [8].

A meta-analysis of 38 studies published between 1967 and March 2009 found that the pooled overall preference rate for c-sections was 15.6% (95% CI 12.5%-18.9) [9]. Different factors can explain this preference. The fear of giving birth (and particularly fear of labour pain) is the most common reason for a woman to request a c-section [2,10,11] and leads to an increase in c-sections on maternal request [7,11–13]. Economic, social, and cultural factors (particularly the influence of the media) and the changing attitudes of women towards c-sections have led women to view this procedure as a way to avoid the unpredictable dangers of childbirth. In addition, clinicians’ attitudes towards c-sections have changed, particularly those of young obstetricians, contributing to the increasing rates of caesarean delivery on maternal request worldwide [2]. In Sweden, women who request c-sections are more likely to judge their health status as poor. Additionally, these women often planned to have only one child and were more likely to report anxiety due to the lack of support during the delivery process, anxiety about the loss of control, and concern regarding the well-being of the foetus [14]. Women giving birth by c-section on maternal request are more likely to have a psychiatric illness [15], and women who have greater exposure to biotechnology or have undergone a medical intervention during their pregnancy (such as in vitro fertilisation or repeated ultrasound scans) are also more likely to request a c-section [12,16].

The aim of this article is to review and summarise recent published studies investigating women’s demand for c-section. We aimed to describe the characteristics, methodologies, country characteristics and country income levels in the studies measuring this c-section demand.

## Materials and methods

We followed PRISMA (Preferred Reporting Items for Systematic Review and Meta-Analysis) Guidelines in conducting this systematic literature review. Under the concept of women’s demand, we mean women’s social demand, and we included women’s preferences and women’s requests. We defined preference for c-section as the choice expressed by a pregnant or non-pregnant woman declaring that she would prefer to give birth by c-section in the future. We defined caesarean delivery on maternal request (CDMR) as any c-section that was requested by the woman and performed in the absence of obstetric or medical indications.

### Article selection

We included research articles that quantitatively measured c-section demand and are based on original data. No restriction was applied regarding the country, study design or methodology. We restricted the search to articles written in English and French.

We excluded articles targeting specific populations (e.g., women with prior c-sections, medically assisted pregnancies, birth practitioners, women exclusively requesting c-section), letters to the editor, editorials, commentaries, committee opinions, duplicate studies, and purely qualitative studies. We performed a quality assessment of the individual studies, focusing on the definitions given for preference and/or CDMR. We did not include articles with unclear definitions, especially those with no clear distinction among elective, planned, and requested c-sections. The first author pre-screened the titles and abstracts of all potentially eligible studies. The full-text versions of all potentially eligible articles were then obtained and assessed to evaluate whether the articles met the inclusion criteria.

### Electronic search

The research focused on the subjects and the types of studies defined in agreement with the authors. We conducted two literature searches.

First, we performed a manual search of websites publishing guidance, technology assessment reports, advice, quality standards and information services and websites of learned societies competent in the field studied. The list of the sites consulted is provided in S1 File.

Second, we carried out a systematic literature search using the Medline database in PubMed. As it appeared to us that women’s demand for c-section became a scientific and global concern beginning in the 2000s, we evaluated articles published between 1 January 2000 and 1 May 2017, in English or French. A professional librarian developed a highly sensitive search strategy. The following full electronic search strategy was used: (“patient preference”[ti] OR ask*[ti] OR request*[ti] OR demand[ti] OR choos*[ti] OR CSMR[tiab] OR CDMR[tiab]) AND (caesarean[tiab] OR cesarean[tiab] OR "Cesarean Section"[Mesh] OR "Elective Surgical Procedures"[Mesh]). This research was completed using the references cited in the documents analysed.

### Analysis

Data regarding the following variables were extracted: region, country, country income level, year of the study, study design, total number of women in the study, total number of c-sections included in the study, total number of women requesting a c-section in the study and/or total number of women preferring a c-section, percentage of c-sections in the study, percentage of CDMR in the study and/or percentage of women preferring a c-section for future births.

Narrative tables were used to describe the study designs, study tools, study location and type and timing of measurements in the articles included in the review.

We calculated the median and interquartile ranges of the number of women included in each selected study to estimate sample sizes using Excel software 2013. For studies that reported data collected during different periods or in different places, we averaged the data in each study. Sample sizes were compared according to the type of study design and outcome using the Wilcoxon rank-sum test. We applied the Word Bank classification to classify countries according to their income per capita in 2017 (https://data.worldbank.org/country, consulted in October 2017).

## Results

**Fig 1:**
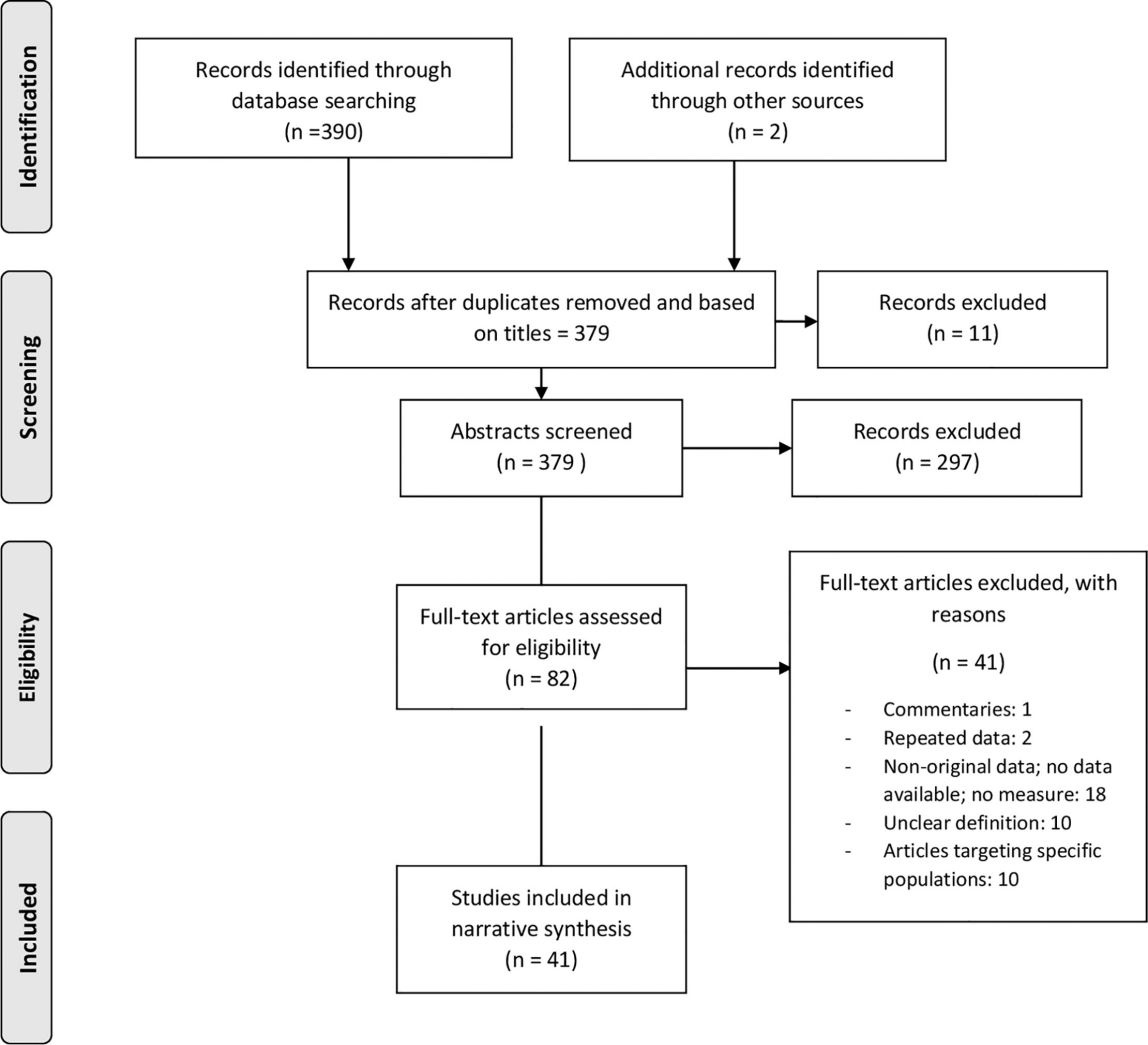
Flow diagram of the search and study inclusion process.

The search strategy retrieved 390 studies. Two additional studies were identified through other sources. After reviewing the titles and removing duplicate studies, 379 studies remained for screening. In total, 297 articles were excluded after reviewing the abstracts, and 82 articles were fully assessed for eligibility. Ultimately, 41 studies met the inclusion criteria and were included in the data collection (Fig 1), representing a total sample of 3 774 458 women.

### Study designs, outcomes and tools

**Fig 2:**
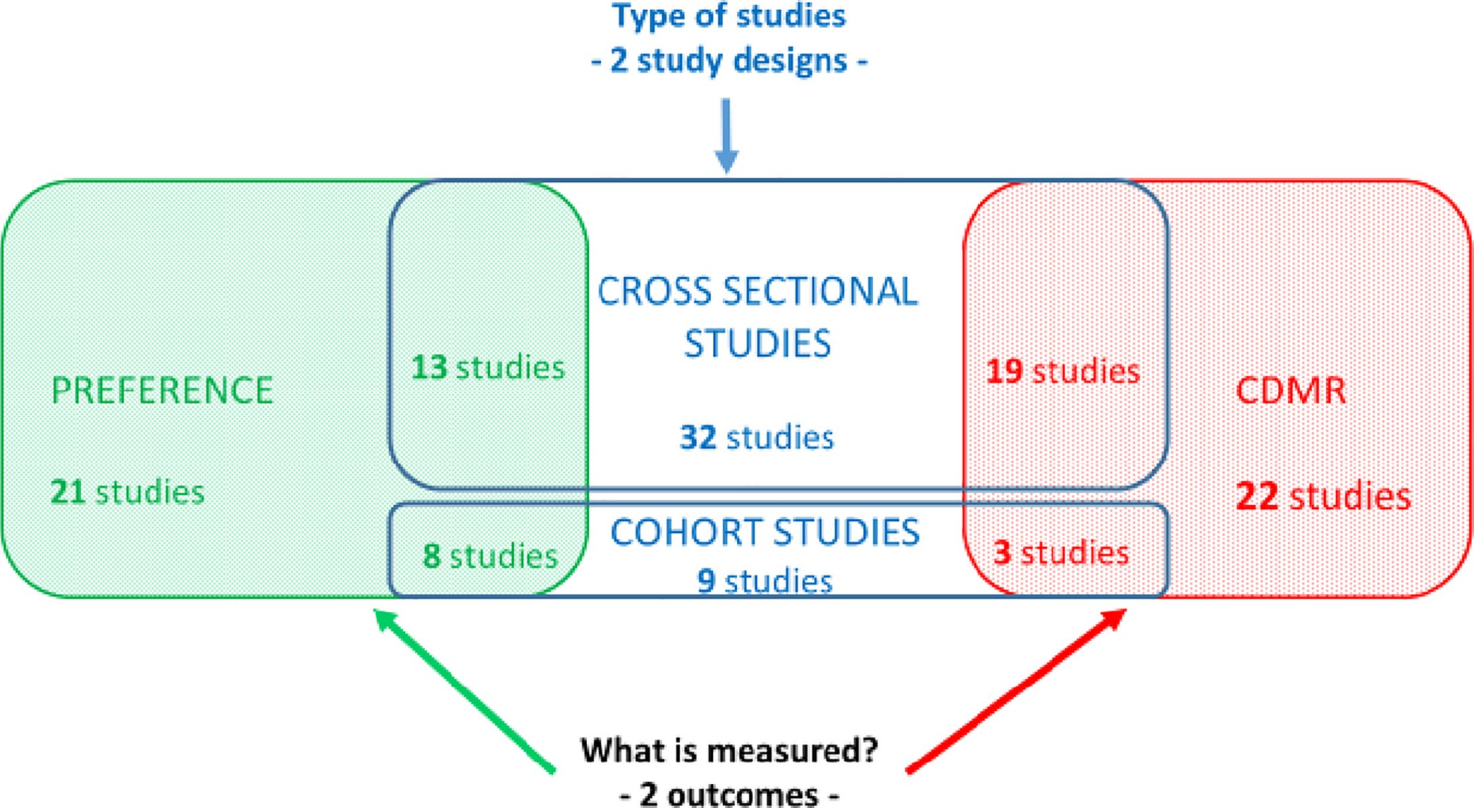
Occurrence of both study designs to measure caesarean demand and outcomes.

We found that cross-sectional studies and prospective cohort studies were the two study designs commonly employed to measure women’s demand for c-sections. Additionally, two different types of outcomes were reported: (i) the preferences of pregnant or non-pregnant women regarding the method of childbirth in the future and (ii) CDMR. Fig 2 displays the frequencies of both the study designs and outcomes.

Table 1 summarises the major characteristics and methodologies used in the included studies. Most studies were recently published, with only eight published before 2010 [17–24], and 33 in or after 2010 [25–57]. Cross-sectional studies (n = 32) [17–20,22,24–28,31–37,41–48,50–56] were more common than were prospective cohort studies (n = 9) [21,23,29,30,38–40,49,57] with at least two different time-points of data collection. Most studies were conducted using self-administered questionnaires (n = 18) [20–22,24,30,37,39–42,44,47,48,52–56]. Fourteen studies used medical record-based questionnaires [18,19,27,28,31–35,43,45,46,50,51], six used interviewer-based questionnaires [23,25,29,38,49,57], and three extracted data from national databases [17,26,36]. Of these studies, 21 evaluated women’s preferences before childbirth [20–22,24,29,30,37–42,44,48,49,52–57], including eight prospective cohort studies [21,29,30,38–40,50,56] and 13 cross-sectional studies [20,22,24,37,41,42,44,48,52–56]. In total, 22 studies assessed whether c-sections were truly performed due to maternal request (CDMR) [17–19,23,25–28,30–36,39,43,45–47,50,51], including three prospective cohort studies [23,30,39] and 19 cross-sectional studies [17–19,25–28,31–36,43,45–47,50,51]. No study measured demand for c-sections during the childbirth process.

**Table 1:** Characteristics and methodologies of the included studies.

* *	*Number of studies*	*Study reference numbers*
**Year of publication**		
**2010 or after**	33	[25–57]
**Before 2010**	8	[17–24]
**Study design**		
**Cross-sectional**	32	[17–20,22,24–28,31–37,41–48,50–56]
**Cohort**	9	[21,23,29,30,38–40,49,57]
**What is measured? (outcomes)**		
**Preference**	**21**	
**Pregnant women *n = 19***		[20–22,24,29,30,37–41,44,48,49,52,53,55–57]
**Non-pregnant women** ***n = 2***		[42,54]
**CDMR**	**22**	[17–19,23,25–28,30–36,39,43,45–47,50,51]
**Source**		
**Self-administered questionnaire**	18	[20–22,24,30,37,39–42,44,47,48,52–56]
**Medical record-based questionnaire**	14	[18,19,27,28,31–35,43,45,46,50,51]
**Interviewer-based questionnaire**	6	[23,25,29,38,49,57]
**Data extracted from a national database**	3	[17,26,36]
**Sample size**		
**More than 100 000 women**	8	[17,26,28,32–34,36,46]
**10 000–100 000 women**	7	[18,31,35,39,43,50,51]
**1 000–9 999 women**	9	[19,23,25,27,40,42,44,45,49]
**500–999 women**	5	[30,47,52,55,56]
**100–499 women**	12	[20–22,24,29,37,38,41,48,53,54,57]
**Less than 100 women**	0	

### Observed c-section preference and request rates

The proportions of women declaring that they would prefer to give birth by c-section ranged from 1.0% (a study in the United Kingdom [21]) to 62.2% (a study in Iran [48]). The proportions of CDMR among all deliveries ranged from 0.2% (a study in Ireland [43]) to 24.7% (a study in China [31]).

### Sample sizes by study design and outcomes

The median and interquartile ranges of the sample sizes used in studies measuring women’s preferences before childbirth were significantly lower than those in studies measuring the CDMR (382, IQR 257–843, *versus* 31 248, IQR 4 115–108 847, p<0.001). The statistical tests did not show any differences in medians or sample size distribution among the studies according to their design (cohorts: median 619, IQR 257–1 789; cross-sectional studies: median 2 992.5, IQR 507.5–84 325).

### Study location and country income levels

Table 2 provides the country characteristics and income levels in the included studies. Most studies were performed in Asia (n = 15) [17,25–38] and Europe (n = 11) [18–21,39–45]. Five studies were conducted in North America [22,51–54], five in the Middle East [46–50], three in Africa [23,55,56], one in Australia [24] and one in Latin America [57]. All included studies were conducted in middle-income (n = 23) [17,23,25–38,46–49,55–57] and high-income countries (n = 18) [18–22,24,39–45,50–54], and we were unable to identify any study performed in low-income countries.

**Table 2:** Country characteristics and country income levels of the included studies.

*Region*	*Number of studies*		
**Asia**	**15**		
**China**		*11*	[25–35]
**Taiwan**		*2*	[17,36]
**Cambodia**		*1*	[38]
**Thailand**		*1*	[37]
**Europe**	**11**		
**Norway**		*3*	[18,39,40]
**Lithuania**		*1*	[41]
**Sweden**		*1*	[19]
**Italia**		*1*	[42]
**Ireland**		*1*	[43]
**Switzerland**		*2*	[20,45]
**United Kingdom**		*1*	[21]
**Multi-countries**		*1*	[44]
**North America**	**5**		
**USA**		*4*	[22,51–53]
**Canada**		*1*	[54]
**Middle East**	**5**		
**Iran**		*4*	[46–49]
**Saudi Arabia**		*1*	[50]
**Africa**	**3**		
**Nigeria**		*3*	[23,55,56]
**Australia**	**1**		[24]
**Latin America**	**1**		[57]
**Country income level**			
**High-income**	**18**		[18–22,24,39–45,50–54]
**Middle-income**	**23**		
**Upper Middle-income**		*19*	[17,25–37,46–49,57]
**Lower Middle-income**		*4*	[23,38, 55,56]
**Low-income**	**0**		
**Total**	**41**		

## Discussion

We found that demand for c-section was measured using two different study designs; demand was measured before pregnancy, during pregnancy, and/or after delivery. Demand for c-section was not measured during the childbirth process. We also found that demand for c-section was measured in high- and in middle-income countries, but demand for c-section was not measured in low-income countries.

### Measuring c-section demand is challenging

We found that c-section demand was measured using the following two different study designs: cross-sectional studies or prospective cohort studies. Furthermore, this demand was measured according to the following two different outcomes: women’s preferences for c-section before delivery and the number of c-sections performed due to women’s requests (CDMR).

#### Cohort and cross-sectional designs

The cohort design allows researchers to track changes in women’s perspectives regarding their willingness to deliver by c-section, while a cross-sectional study captures this demand only at a specific point of time. Using a cohort design, women can be interviewed several times during before their pregnancy, during their pregnancy, and before and after childbirth. This study design highlights changes in women’s preferences over time. Indeed, feelings regarding childbirth can change over time. According to a study conducted in the United Kingdom, knowledge acquisition by women regarding birth preferences begins before pregnancy and is a dynamic process. Women accumulate knowledge from different sources until an informed choice can be made [21]. In China, although most women do not initially want to deliver surgically, many women ultimately deliver by c-section. This change may occur during the third trimester of pregnancy or during labour. Doctors play an important role in the decision to undergo a c-section without a medical indication [30]. Another study in China using a prospective design showed that the preference for c-section among nulliparous births significantly increased from 10.1% during pregnancy to 28.0% after childbirth [29], indicating that childbirth is a stressful experience. Therefore, a cohort study design allows for a deeper analysis of women’s perspectives regarding their preferred method of childbirth and the requests made by women.

#### Preference and CDMR

Both outcomes used to measure c-section requests have limitations. Measuring women’s preferences for c-sections before or during pregnancy is interesting, but we cannot ensure that women who prefer a c-section delivery will actually ask for a caesarean delivery at the appropriate time. Regardless, the preference for a c-section and subsequent delivery by c-section are strongly linked [29,39,58].

Furthermore, measuring CDMRs using a medical record-based questionnaire after childbirth is challenging. Maternal requests can often be omitted by obstetricians who prefer to mention a medical indication for a c-section in the medical record. For example, in countries such as France, women’s requests are likely underestimated in medical files, and obstetricians tend to report a medical indication to protect themselves legally [59]. In other contexts where obstetricians feel pressure to justify high c-section rates, providing the indication ‘maternal request’ shifts the responsibility from the obstetrician to the woman [4].

Additionally, measuring CDMRs with self-administered or interviewer-based questionnaires is challenging. Excluding a concomitant medical issue in women who report that they requested a c-section is difficult because these women may have ignored or omitted the medical indication. Post hoc rationalisation by women after delivery has also been reported [4].

### The social construction of c-section demand

In our systematic literature review, demand for c-section was measured before or after delivery. We hypothesise that certain major determinants involved in the social construction of c-section demand are unknown in the different methods reported thus far in the literature. Indeed, two determinants, i.e., the influence of biomedicine in the process of childbirth and women’s experiences inside the delivery room, play a major role that may interfere and be decisive factors in women’s preferences during pregnancy and c-sections actually performed by maternal request.

#### The biomedical impact

Wagner explains that doctors promote women’s right to choose c-sections because c-sections are a doctor-friendly procedure [6]. Encouraging women to choose a c-section is suitable for doctors. Hopkins also notes that the power structure between women and doctors is unequal [7]. In the decision-making process, the authority of doctors can clearly influence women, depending on the manner in which the doctor presents the benefits and risks of c-sections, which might lead women to “choose” a c-section. This structural asymmetry disempowers women and is particularly relevant during the childbirth process and inside labour wards, where women may be particularly vulnerable.

According to a large study, the diagnosis of nuchal cord using ultrasound is the single most frequent reason for a c-section in China [27]. This non-medically justified indication has also been frequently reported in Cambodia [38]. This trend illustrates how biotechnology (in this case, ultrasound) can influence the decision-making of women during pregnancy and contribute to a social demand for c-section. Women who have been informed by practitioners that their child has a nuchal cord may be afraid and pressed by the medical institution to request a c-section. In this way, childbirth can be constructed as a fearful event [10]. Structural violence—defined by the subtle and invisible ways in which a social institution (in this case, the medical institution) perpetuates social inequity (in this case, gender inequity)—is transmitted to women and expressed as a caesarean request. In a medicalised context, women are losing confidence in their ability to give birth naturally [10]. This lack of confidence, combined with a poor environment of quality of care, leads these women to develop strategies to survive in an unsupportive environment.

#### Poor support during the childbirth process and structural violence

A lack of agreement regarding the definition of c-section by maternal request exists in the literature. A review of 105 articles attempted to establish a clear definition of c-section by maternal request [60]. The authors concluded that CDMR might have the following two characteristics according to the existing literature: i) a CDMR is performed *before* the onset of labour, and ii) it is performed in the absence of a medical indication. The authors noted that all studies agreed on the timing component, though only one study emphasised that investigating c-section requests during the childbirth process is important.

We agree that c-section requests *during the childbirth process* must be assessed. In our systematic review investigating the measurement of women’s demand for a c-section, we were able to identify studies assessing the preference of women before the onset of labour and studies assessing c-section effectively performed on maternal request (CDMR). But we did not find a study that evaluated women’s preference before and after the beginning of labour.

A recent study investigating c-section requests during the childbirth process was conducted in Cambodia [38] and showed that pain and fear inside labour wards drive women to request c-sections during labour. The few hours inside the labour wards are rarely investigated; however, this time period appears to be critical for c-section requests, particularly in countries in which neither epidural anaesthesia nor continuous support during childbirth are available. China has a high rate of c-sections, and most studies included in our review were conducted in China (n = 11 studies). Similar to Cambodia, China lacks effective pharmacological pain relief or social support during labour, and many women consider c-sections to be a way to mitigate and avoid labour pain [61]. The lack of studies measuring maternal requests during labour in our systematic review suggests that the suffering, pain and distress women experience in labour wards are often disregarded.

This structural violence must receive attention from policy makers to decrease the mistreatment of women. Mistreatment of women during childbirth in health facilities occurs not only at the level of the interaction between the woman and the provider but also through systemic failures at the health facility level [62]. Requests for c-sections voiced in the delivery room are driven by pain and fear and must be captured at this precise time. This request inside the labour room is not documented by classical surveys (i.e., demographic and health surveys) or common survey designs (i.e., medical record-based questionnaire, national databases). This request must be quantitatively measured by questionnaires specifically focusing on this point and by constructing specific relevant indicators. Researchers should evaluate women’s preference before and after the onset of labour and link this apparent contradiction with the condition supported by women during the delivery process. Studies of this nature may allow us to link the social construction of maternal demand for c-sections with structural violence.

### Low-income countries: Determining caesarean demand

Women’s rates of request were measured in high- and in middle-income countries. We found no studies measuring maternal demand for c-section in low-income countries. In our systematic review, the highest rate of women preferring a c-section was observed in Iran, which is a middle-income country where 62.2% of women declared during their pregnancy that they would prefer to give birth by c-section [48]. The highest rate of CDMR was found in China, which is also a middle-income country, and in one study, 24.7% of all deliveries were via c-section performed on maternal request [31]. A previous meta-analysis similarly concluded that middle-income countries had higher preferences for c-sections, and no studies from low-income countries were included in that meta-analysis [9]. It is unclear why the rates of maternal demand for c-section have not yet been quantitatively investigated in low-income countries.

Nigeria, classified as a lower middle-income country, is the only country in sub-Saharan Africa in our review where studies on c-section requests have been conducted. In Nigeria, poor quality of care has been reported to be a leading factor for c-section requests by mothers because epidural anaesthesia and monitoring of the childbirth process are largely unavailable [55]. In low-income countries, the degree of poor quality of care is substantial, and authors have identified that this deficit is leading women to request c-sections without a medical indication [8]. Continuous support has proven to be effective, including leading to fewer c-sections [63]. Supportive care for women during childbirth and adequate pain relief (including non-pharmacological pain management), along with appropriate technology, must be available for each woman in all locations.

More studies investigating maternal demand for c-section are needed in low-income countries where the private sector is quickly developing, providing opportunities for the upper and middle classes to develop a preference for c-sections to avoid poor quality of care during childbirth. A lack of confidence in the quality of obstetric care may increase CDMRs [55], and places perceived by women as dangerous due to poor quality and safety may encourage women to request a c-section [8]. Due to this particular context, we consider these c-section requests in low- and middle-income countries with poor quality of care as *destress c-sections*.

### Limitations

This study has certain limitations. First, only one reviewer screened the articles and extracted the data due to human resources, timing and funding constraints. Second, only one database was searched. Third, we included studies written in English and French only. Including studies written in Spanish and Portuguese may have made this work more comprehensive and would have allowed for the inclusion of more studies conducted in Latin America. Brazil and Mexico are characterised by highly medicalised births, and c-section determinants have been extensively studied in these countries. Finally, we did not perform a meta-analysis of women’s preference and CDMR rates.

## Conclusion and recommendations

Driven by continually increasing c-section rates worldwide, studies have recently been conducted to measure women’s demand for c-section. However, certain determinants remain unknown. We recommend establishing prospective cohort studies to develop a holistic approach to determining women's preferences and requests for their delivery mode. The social construction of caesarean section must be investigated, and the structural violence in which many women are delivering, without proper pain management and/or social support, must be exposed. We also recommend establishing studies in low-income countries where demand for c-section is rarely considered, and details regarding this demand are unknown.

## Supporting information

S1 File List of consulted websites during the literature search. (DOCX)

S2 File PRISMA checklist. (DOCX)

S1 Dataset (XLSX)
