# Supplementary material for: How is women’s demand for caesarean section measured? A systematic literature review
Source: PLoS One. 2019 Mar 6;14(3):e0213352. doi: 10.1371/journal.pone.0213352 (PMC6402700; doi:10.1371/journal.pone.0213352)
Supplement: S1 File — (DOCX) [file pone.0213352.s001.docx]

**S1 File**

List of consulted websites during the literature search:

- Association Canadienne des sages-femmes
- Catalogue et index des sites médicaux francophones – CISMeF
- Centre de ressources et d'expertise pour l'enfant – ASBL
- Centre d’expertise et de référence en santé publique – INSPQ
- Centre Fédéral d’expertise - KCE
- Collège National des Gynécologues et Obstétriciens Français – CNGOF
- Collège National des Sages-Femmes – CNSF
- Direction de la recherche, des études, de l’évaluation et des statistiques – DREES
- Institut National de Prévention et d’Éducation pour la santé – INPES
- Ministère des affaires sociales et de la santé
- Organisation Mondiale pour la Santé – OMS
- Société des obstétriciens et gynécologues du Canada - SOGC
- American Congress of Obstetricians and Gynecologists – ACOG
- Association of Ontario Midwives Australian and New Zealand Stillbirth Alliance – ANZSA
- Centre for Reviews and Dissemination databases
- Childbirth Connection
- CMA Infobase
- Cochrane Library
- Euroscan
- Institute for Clinical Systems Improvement – ICSI
- Institut of medicine - IOM
- International confederation of midwives National Childbirth Trust - NCT
- National Guideline Clearinghouse – NGC
- National Health Services Evidence
- National Institute of Child Health and Human Development Maternal Fetal Medicine Units Network
- National Institute for Health and Care Excellence – NICE
- National Institute for Health Research – NIHR
- Queensland Maternity and Neonatal Clinical Guideline
- Royal College of Obstetricians and Gynaecologists – RCOG
- Royal College of Midwives - RCM
